# Preferential regulation of stably expressed genes in the human genome suggests a widespread expression buffering role of microRNAs

**DOI:** 10.1186/1471-2164-13-S7-S14

**Published:** 2012-12-07

**Authors:** Zhen Yang, Dong Dong, Zhaolei Zhang, M James C Crabbe, Li Wang, Yang Zhong

**Affiliations:** 1 School of Life Sciences, Fudan University, Shanghai, 200433, China; 2 Institute of Molecular Ecology and Evolution, iAIR, East China Normal University, Shanghai, 200062, China; 3 Donnelly Centre for Cellular and Biomolecular Research, University of Toronto, 160 College Street, Toronto, ON, M5S 1A8, Canada; 4 Institute of Biomedical and Environmental Science & Technology, Faculty of Creative Arts, Technologies and Science, University of Bedfordshire, Luton LU1 3JU, UK; 5 Shanghai Center for Bioinformation Technology, Shanghai 200235, China; 6 Institute of Biodiversity Science and Geobiology, Tibet University, Lhasa, 850000, China

## Abstract

**Background:**

MicroRNAs (miRNAs) are a class of small noncoding RNAs that regulate the target gene expression at post-transcriptional level. They are widely involved in biological processes, such as embryonic development, cell division, differentiation, and apoptosis. Evidence suggests that miRNAs can constrain the variation of their target to buffer the fluctuation of expression. However, whether this effect can act on the genome-wide expression remains controversial.

**Results:**

In this study, we comprehensively explored the stably expressed genes (SE genes) and fluctuant genes (FL genes) in the human genome by a meta-analysis of large scale microarray data. We found that these genes have distinct function distributions. miRNA targets are shown to be significantly enriched in SE genes by using propensity analysis of miRNA regulation, supporting the hypothesis that miRNAs can buffer whole genome expression fluctuation. The expression-buffering effect of miRNA is independent of the target site number within the 3'-untranslated region. In addition, we found that gene expression fluctuation is positively correlated with the number of transcription factor binding sites in the promoter region, which suggests that coordination between transcription factors and miRNAs leads to balanced responses to external perturbations.

**Conclusions:**

Our study confirmed that the genetic buffering roles of miRNAs can act on genome expression fluctuation and provides insights into how miRNAs and transcription factors coordinate to cope with external perturbation.

## Background

One of the most remarkable features of biological systems is their inherent robustness against external perturbations. Living systems are continuously confronted with a variety of outside stimuli, such as nutrition, toxins, temperature and humidity. These external inputs must be properly processed to reach a relative self-stability and stability in the output. To achieve this, there must be certain buffering mechanisms to compensate for the genetic or environmental perturbation. For example, gene expression in the cell is rigorously regulated in response to external signals. These genes should be constrained or "canalized" in their expression to an appropriate level. On the other hand, genes have different expression patterns under various biological and environmental conditions; they present different degrees of sensitivity to external perturbation. The expression of many genes is considered robust as they are relatively stable upon perturbations. How this is achieved, i.e. the genetic buffering mechanisms that mediate the stability and robustness are largely unknown. It is suggested that negative feedback loops within regulatory networks serve to buffer expression variation and reduce expression noise in the cell [1]. Also, specific genes could play a role in canalizing gene expression, such as the zygotic gap genes including kruppel and knirps in *Drosophila *[2]. However, it is still largely unclear whether there are any canalizing/buffering mechanisms acting on the genome wide expression.

MicroRNAs (miRNAs) are endogenously expressed small (typically 18-23 nt in length) noncoding RNAs that regulate gene expression at the post-transcriptional level [3,4]. By binding to the 3'-untranslated regions (3'-UTR) of target mRNAs, miRNA can block the expression of their target genes through translational repression or mRNA degradation [5]. miRNA-mediated gene expression regulation is widespread in eukaryotes. A single miRNA can regulate up to several hundred genes, and it is speculated that more than one-third of the genes in the human genome are miRNA targets [6]. Considering the prevalence of miRNA mediated gene expression regulation in mammalian cells, it is fascinating to inquire whether these small ncRNAs can serve as genetic factors that buffer whole genome expression. This hypothesis has been supported in several studies. For example, miR-17 can function in an incoherent feed-forward loop to buffer the translation of E2F1, which is activated by c-Myc [7]. Another evolutionarily conserved miRNA, miR-7, could act in some interlocking feedback and feed-forward loops to confer network stability against perturbation. The miR-7 mediated network is essential for buffering the gene expression variation resulting from temperature fluctuation in *Drosophila *[8].

In addition, some studies have also used bioinformatics tools to investigate the influence of miRNAs on gene expression fluctuation. Cui *et al. *suggested that miRNAs could decrease the cross-species expression divergence and constrain the evolutionary expression variation [9]. Another study indicated that miRNA targets are enriched in duplicated genes, which could be a mechanism for buffering the gene expression variation resulting from whole genome duplication [10]. However, it was suggested that on the population level, miRNAs could increase gene expression variability [11], and Wu *et al., *indicated that miRNA targets are enriched in environmental chemical regulated genes, which have a more variable expressed pattern than others [12]. This controversy likely results from the scales used in different studies and the data sets used, which indicated that a systematic study of this issue is required.

We therefore explored the stably expressed genes (SE genes) and fluctuant genes (FL genes) by comprehensive investigation of mRNA expression profiling data under various environmental conditions. We found that these two groups of genes have a very distinct function distribution. By evaluation of the propensity of miRNA regulation, we found that miRNA targets are significantly enriched among SE genes. This effect is independent of the number of regulatory mRNAs but is relevant to their 3'-UTR length. These observations indicated that miRNAs can play a genetic buffering role to confront genome wide expression fluctuation.

## Results

### Functional enrichment of SE and FL genes

To inspect the influence of miRNAs on gene expression fluctuation, we first conducted a comprehensive analysis of microarray data to retrieve the SE genes and FL genes. We collected the expression profiles under various environmental conditions based on the HGU133plus2.0 platform. To minimize variation caused by different experimental platform, we only investigated expression data generated from this platform. For each gene, a fluctuant score (FL score) was calculated by meta-analysis to quantify the expression sensitivity in response to environmental perturbations. The top and bottom 5% of genes in the list were defined as SE genes and FL genes respectively. To evaluate the validity of this categorization, we performed Gene Ontology (GO) enrichment analysis on these genes [13]. From the resultant GO graph, we observed a distinct function distribution for these two groups of genes (see Additional File 1 and Additional File 2). Specifically, for "molecular function", the SE genes were enriched in terms of some basic activities, such as RNA binding, protein binding, NADH dehydrogenase activity, constituent of the ribosome etc, whereas FL genes are involved in environmental factor response, such as receptor binding, cytokine activity, growth factor receptor binding, peptide hormone binding and dopamine binding. For "biological processes", the SE genes were enriched in translation, gene expression, metabolic processes, and biosynthetic processes, whereas FL genes were enriched in signaling pathways, defence response, regulation of immune system process and mediation by a chemical signal etc. Similar results were also obtained when the top and bottom 10% of genes were defined as SE genes and FL genes. This suggests that our classification of SR and FL genes are biologically meaningful and these genes occupy distinct positions in the cell.

### miRNA targets are preferentially enriched in SE genes

**Figure 1:**
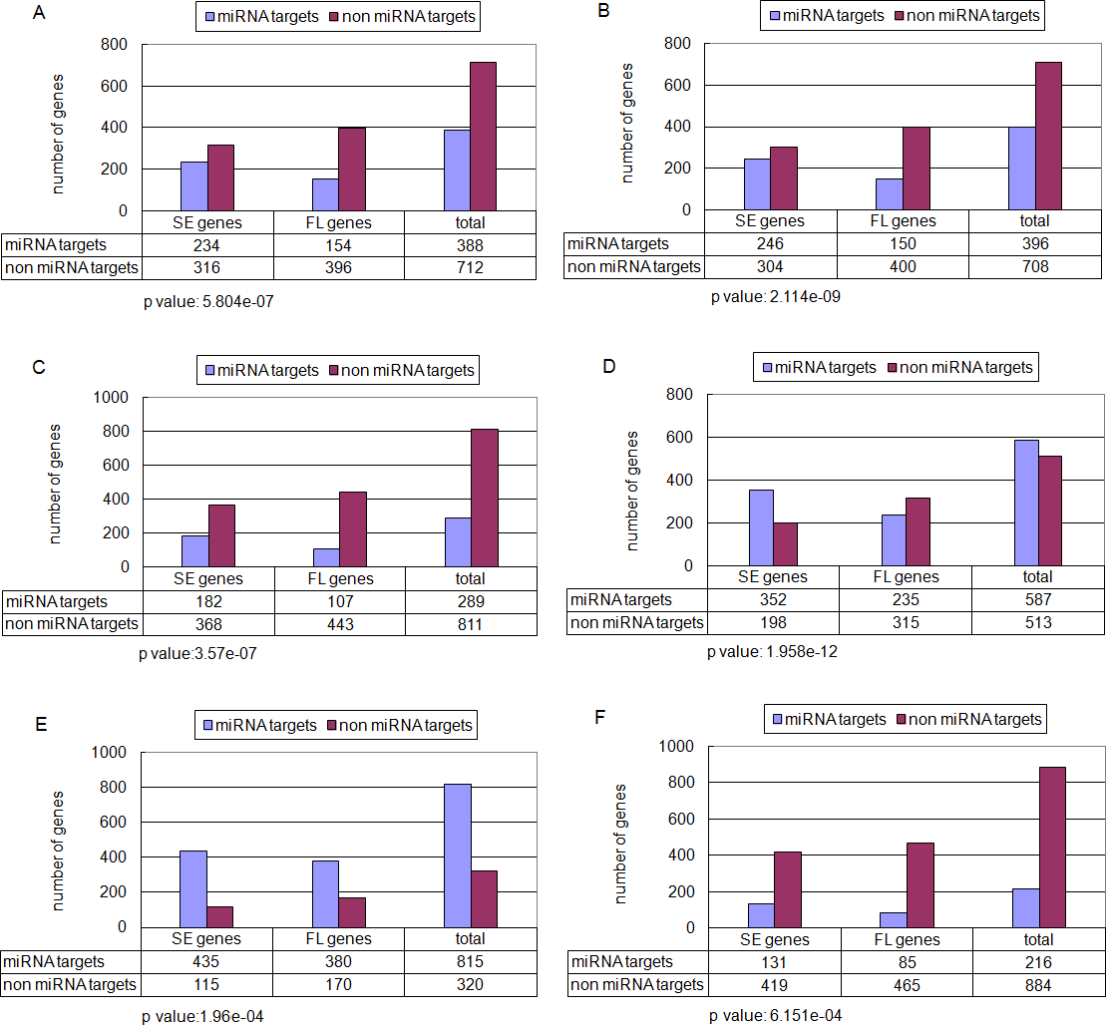
**miRNA targets are enriched among SE genes**. This figure shows the number of miRNA targets and non-miRNA targets among SE genes and FL genes (A) predicted by PicTar, (B) predicted by TargetScan, (C) predicted by both PicTar and TargetScan (intersections), (D) predicted by PITA, (E) predicted by miRanda and (F) by experimentally validation. The top and bottom 5% of the gene were defined as SE genes and FL genes respectively.

We evaluated the propensity of miRNA regulation based on the SE gene and FL gene classification scheme. The predicted targets of human miRNAs were retrieved from TargetScan [6], PicTar [14], PITA [15] and miRanda [16,17], which to our knowledge are regarded in the community as having higher prediction accuracy. A more stringent prediction result derived from intersection of TargetScan and PicTar provided by the miRGen database was also used [18]. In addition, another set of experimentally validated miRNA targets integrated from miRTarBase [19], miRrecords [20], miRWalk [21] and miR2Disease [22] was also included in this analysis. Based on these data sets, we observed that miRNA targets were significantly enriched in SE genes. As shown in Figure 1A, miRNA targets comprised 42.5% of SE genes, but only 28% of FL genes as predicted by PicTar (Fisher exact test p-value = 5.8e-07). We observed similar results when using the data sets from other algorithms and experimentally validated miRNA targets (Figure 1B-F). As a control, we randomly selected the same number of genes from the list to analyze this trend, no obvious propensity of miRNA regulation was found in the control data sets (see Additional File 3). The propensity of miRNA regulation was also observed when we selected the top and bottom 10% of the genes as SE genes and FL genes respectively (see Additional File 4). Furthermore, to exclude the interference of datasets from cancer tissue or cell lines, we selected 69 microarray datasets that were derived only from normal tissues to screen the SE genes and FL genes. The propensity analysis of miRNA regulation gave similar results (see Additional File 5).

**Figure 2:**
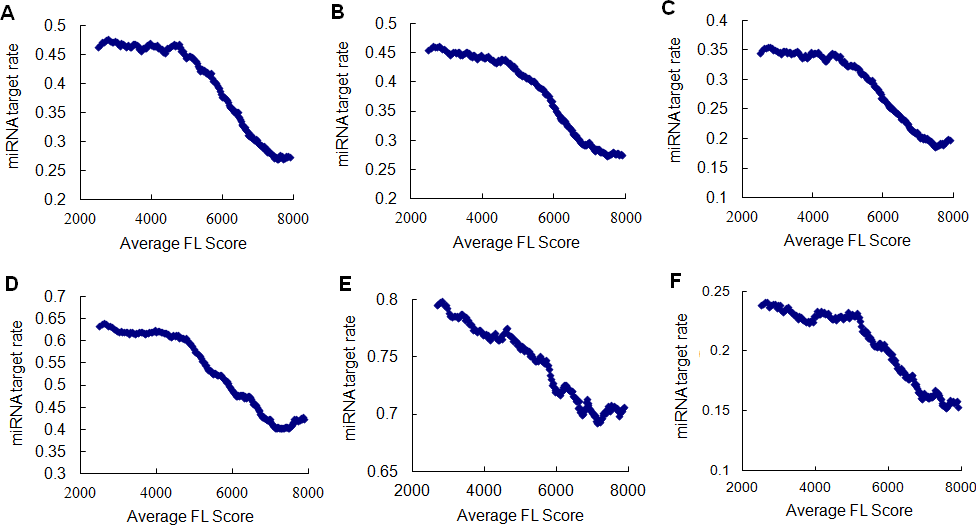
**Proportion of miRNA target among genes with different average FL scores**. (A) miRNA targets predicted by PicTar among genes with different FL scores, Pearson correlation coefficient r = -0.94, p value = 1.91e-87; (B) miRNA targets predicted by TargetScan among genes with different FL scores, Pearson correlation coefficient r = -0.96, p value = 1.05e-104; (B) miRNA targets predicted PicTar and TargetScan among genes with different FL scores, Pearson correlation coefficient r = -0.95, p value = 5.73e-94; (D) miRNA targets predicted PITA among genes with different FL scores, Pearson correlation coefficient r = -0.96, p value = 7.06e-106. (E) miRNA targets predicted miRanda among genes with different FL scores, Pearson correlation coefficient r = -0.97, p value = 8.25e-117. (F) experimentally validated miRNA targets among genes with different FL scores, Pearson correlation coefficient r = -0.94, p value = 5.30e-90.

To avoid potential bias derived from sampling, we next divided the total genes into two groups and calculated the average FL score in each group. The first group contained all the predicted miRNA target genes whereas the second group contained the other genes. We found that the expression fluctuation of miRNA target genes was significantly lower than that of the non-miRNA-target genes for the four data sets (Table 1). For example with PicTar, the average FL score of miRNA target genes was 5154.0, significantly lower than the non-miRNA targets (average FL score = 5717.3, Wilcoxon rank sum test, p-value: 3.53e-58). For a more detailed analysis, we subgrouped the total genes according to their FL scores, and calculated the average FL score and miRNA target proportion in each group. As shown in Figure 2, there was a negative correlation between expression fluctuation and miRNA target proportion, and miRNA target proportion declined dramatically with increasing FL score. Taken together, these results indicated that miRNA target genes are significantly enriched in SE genes, which suggests that miRNAs have a negative effect on whole genome expression fluctuation.

**Table 1:** Average FL score and standard deviations of miRNA targets and non-miRNA targets

	miRNA targets	Non-miRNA targets	p-value
PicTar	5154.08 ± 1867.46	5717.35 ± 1891.43	3.53E-58
TargetScan	5150.78 ± 1899.46	5707.21 ± 1873.56	8.62E-53
P & T Intersection	5103.05 ± 1881.42	5654.68 ± 1887.80	6.68E-46
PITA	5191.96 ± 1919.22	5839.51 ± 1824.66	9.83E-74
miRanda	5415.45 ± 1913.18	5752.54 ± 1847.57	6.63E-17
Validated	5203.18 ± 1890.85	5576.93 ± 1897.61	4.86E-18

Average FL score of miRNA targets is significantly lower than that of non-miRNA targets, the p-value was drawn from Wilcoxon rank sum test.

### Gene expression fluctuation buffering is independent of the number of regulatory miRNAs

Several studies have demonstrated that a single miRNA can regulate hundreds of mRNAs and that a single mRNA can be regulated by multiple miRNAs. This complex interaction makes the synergistic effect of miRNA regulation in biological networks and pathways possible [23,24]. The synergistic effect of different miRNAs on the expression level of a single gene has been reported [25]. However, whether this effect exists on the genome-wide level is largely unknown. We therefore analyzed the correlation between number of regulatory miRNAs in the 3'-UTR and gene expression fluctuation. In the following analysis, we only use the predicted miRNA targets from PicTar, TargetScan and PITA in that too large or too small data sets may introduce interference. Predicted miRNA target genes were subgrouped according to the number of regulatory miRNAs within 3'-UTR and then the average FL score in each group was calculated. We did not observe any significant correlation between the number of regulatory miRNAs and the expression fluctuation (see Additional File 6). This result is somewhat in disagreement with the previously reported positive correlation between gene expression variability and miRNA seed number [11]. To account for such disagreement, we propose that following explanation. On one hand, a gene that is regulated by multiple microRNAs may be an indication of its functional importance, which requires complex post-transcriptional control by miRNAs. Such functional importance suggests that the expression of such genes are tightly controlled and has less variations. On the other hand, such sophisticated regulation by multiple miRNAs may render it prone to fluctuations and accumulation of noise. We believe that these two factors may be both in play for the majority of the miRNA target genes, and for any given gene it is uncertain which factor is more dominant. As a result, we do not expect any straightforward and overwhelming correlation between the gene expression fluctuation level and the number of miRNA seeds.

### Gene expression fluctuation and 3'-UTR length

**Figure 3:**
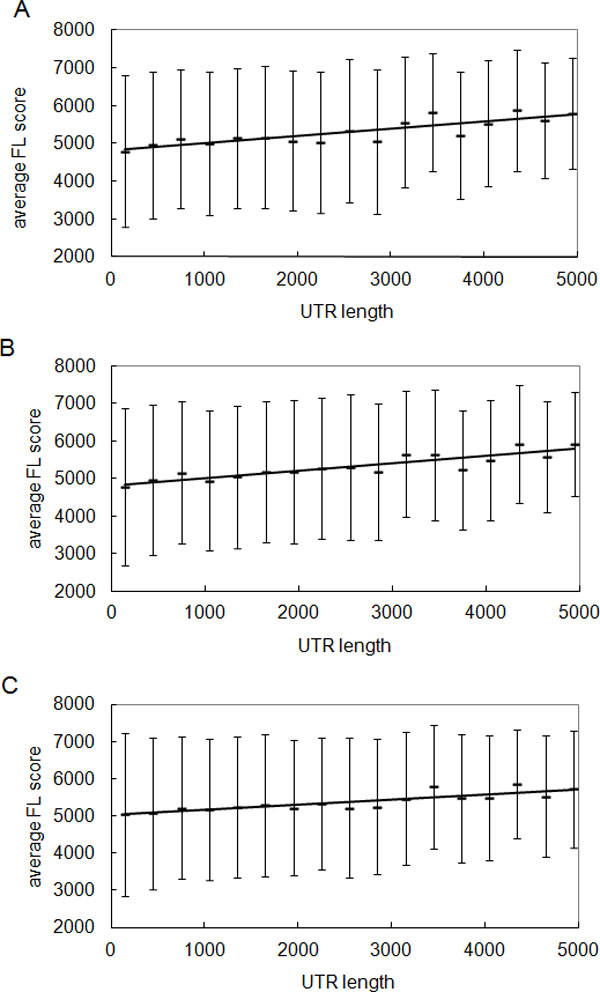
**Correlation between gene expression fluctuation and 3'-UTR length**. Positive correlation between expression fluctuation and 3'-UTR length was observed. (A) average FL score and 3'-UTR length from PicTar predicted miRNA targets, Pearson correlation coefficient, r = 0.85, p value: 1.69e-05. (B) average FL score and 3'-UTR length from TargetScan predicted miRNA targets, Pearson correlation coefficient, r = 0.89, p value: 2.01e-06. (C) average FL score and 3'-UTR length from PITA predicted miRNA targets, Pearson correlation coefficient, r = 0.86, p value: 7.31e-06.

**Figure 4:**
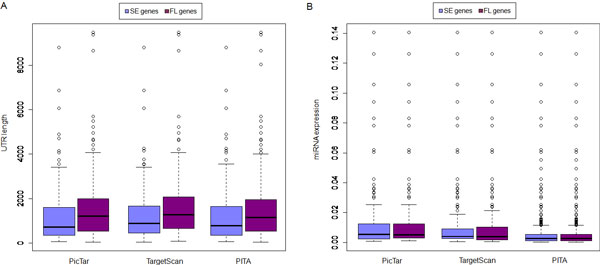
**UTR length of miRNA targets and expression intensity of miRNAs that regulate SE and FL genes**. (A) 3'-UTR length of predicted miRNA targets in SE genes is shorter than that of FL genes. (B) No significant difference between average expression intensity of miRNAs that regulate SE genes and FL genes.

Most of the miRNA target sites are located in the 3'-UTR of mRNAs, whereas the lengths of 3'-UTR of protein coding genes vary substantially, and it has been shown that miRNA regulation has an effect on 3'-UTR evolution. It is also known that genes with different 3'-UTR lengths have distinct expression patterns [26,27]. Along this line, we performed a correlation analysis between 3'-UTR length and gene expression fluctuation. Predicted miRNA targets were subgrouped by length in 300 nt intervals and the average FL score within each group was calculated. As shown in Figure 3, a positive correlation between expression fluctuation and 3'-UTR length was observed. The average FL score increased with the 3'-UTR length, for example among the PicTar prediction results (Figure 3A), r = 0.85, p value = 1.69e-05. Similar results were obtained when using miRNA targets predicted by TargetScan (Figure 3B) and PITA (Figure 3C). This result was confirmed when we directly compared the 3'-UTR length of miRNA targets in both SE genes and FL genes. We found that the 3'-UTR length of miRNA targets in SE genes was shorter as compared to that of the FL genes (Figure 4A), which suggested that miRNA targets with longer 3'-UTR length were more likely to have higher expression fluctuation, thus other confounding factors may interfere with the gene expression.

To investigate whether the expression intensity of miRNAs have an effect on target expression fluctuation, we obtained the miRNA expression data from micorRNA.org database [17] and calculated the average expression level in 31 normal human tissues. We compared the average expression intensity of miRNAs that regulate SE genes and FL genes. We did not find any significant difference between these groups (Figure 4B), indicating that miRNA expression level is not a decisive factor for target expression fluctuation.

## Discussion

Human genes have different expression patterns and sensitivity in response to external environment perturbations, thus the global analysis of miRNAs on whole genome expression has drawn much attention recently. In this study, we conducted a large scale meta-analysis to explore the genes with different degrees of expression fluctuations. The Gene Ontology enrichment analysis revealed that the stably expressed genes and fluctuant genes have distinct functional categories. Stably expressed genes are mainly involved in basic and essential biological processes and the fluctuant genes are mainly involved in processes in response to external signals. We found that miRNA targets were significantly enriched in stably expressed genes relative to fluctuant genes, suggesting that miRNAs act on the genome-wide expression to reduce their fluctuation. In addition, we found that the gene expression buffering effect was independent of the number of miRNA target sites within the 3'-UTR. However, expression fluctuation was correlated with the 3'-UTR length; and this could result from alternative polyadenylation signals or cis-acting elements other than miRNA binding [28,29]. To explore the miRNAs that play an important role in gene expression buffering, we counted the number of targets for each miRNA in both SE genes and FL genes and investigated whether the targets are more enriched or specific in SE genes or FL genes. According to their preference of regulation, miRNAs were classified as SE gene-related miRNAs (SE-miRNA) or FL gene-related miRNAs (FL-miRNA) (see Additional file 7). Interestedly, we found that the number of SE-miRNAs is greater than that of FL genes, which is consistent with the previous observation.

Our work provides some important insights into the functions of miRNAs. MiRNAs have been postulated to play a dual role in regulating gene expression, i.e. to regulate the mean of the expression output and to modulate the expression variation [30-32]. On one hand, miRNAs can regulate the expression level of critical genes during animal development, which make them indispensable for the survival and normal growth of the cell, and thus evolutionarily conserved [33-37]. On the other hand, many miRNAs are believed to preferentially regulate ubiquitously-expressed genes other than tissue-specific genes [38], and in most cases they only have moderate effect on the mean expression level of the targets as their primary function is to minimize the expression fluctuation in different tissues and in different conditions [39].

As a part of the expression regulatory network, miRNAs are suggested to be involved in mechanisms such as feedback loops and feed-forward loops. Within these mechanisms, miRNAs can cooperate with transcription factors to balance the outputs of their target [40,41]. The expression level of transcription factors are known to be stochastic, which could induce very high level of noise in the regulatory network, and could be detrimental to the cell. The expression buffering role of miRNAs could beneficial to the organism to minimize such noise.

**Figure 5:**
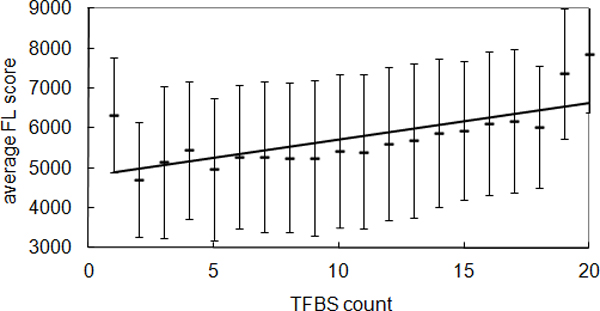
**Correlation between TFBS count and expression fluctuation**. Positive correlation between TFBS count and average FL score was observed, Pearson correlation coefficient, r = 0.72, p value: 3.78e-04.

Motivated by this hypothesis, we obtained the transcription factor binding sites (TFBS) that were previously identified by Xie *et al., *[42] and the promoter sequences from UCSC genome browser [43]. As shown in Figure 5, we found a positive correlation between the number of TFBS and average FL scores, which indicated that TFs could contribute to the regulatory complexity. This result is also consistent with the observation that miRNAs preferentially regulate genes with high transcriptional regulation complexity [44]. These observations suggest that the coordination of TFs and miRNAs in complex networks lead to the internal stability in gene expression of the cell.

## Conclusions

It was hypothesized previously that miRNA mediated regulation can confer expression stability and robustness of their target genes. In this paper, our systematic study provided evidence that miRNAs can buffer expression fluctuation of many human genes. Interestingly we found such effect to be independent from the number of miRNA target sites per gene. We further show evidence that coordination between miRNAs and transcription factors could result in the stability of transcriptional regulatory networks.

## Methods

### Data collection and preprocessing

For identification of the SE genes and FL genes in human genome, firstly we collected gene expression data sets based on the standard and widely-used Affymetrix HGU133plus2.0 platform from the Gene Expression Omnibus database [45]. We collected expression profiles that consist of samples under a variety of environmental factors, including hypoxia, hyperthermia, smoking, alcohol, medicine, strong magnetic field, metal ion, small-sized compounds, chemotherapy, UV, etc. Only data sets with more than six arrays were retained. Finally, a total of 149 data sets were obtained. These data sets were classified as from normal tissues, cancer tissue or cell lines and other disease (see Additional File 8). For each data set, the expression values were logarithmically transformed (base 2) if it was above 0, otherwise turned to 0. Only the maximum expression value was selected if there were multiple probes for a given gene in each sample.

### Identification of SE genes and FL genes

Identification of SE genes and FL genes was performed according to the method previously described by Hao *et al. *with minor modifications [46]. Briefly, the coefficient of variance (CV = standard deviation/mean value) of the expression for each gene in every data set was calculated. Due to the heterogeneity of the data sets, the CVs of specific genes from different data set could not be compared directly. Thus the CVs in each data set were rank ordered in ascending order, to generate a ranked CV matrix. For each gene, the FL score was defined as the average rank order of the CV in the matrix, and was used as the indication of expression fluctuation. For a specific gene, a relatively high CV was expected if it was more vulnerable to the perturbation of environmental factors. Its confidence was deemed higher if this trend was observed in multiple data sets, thus relative high FL score were expected, and vice versa. Based on this hypothesis, the genes occupying the top or bottom of the genes list were taken as the SE genes and the FL genes respectively (presented as Additional File 9). To validate this classification, Gene Ontology enrichment analysis was used to investigate the functional difference between SE genes and FL genes, performed using the hypergeometric test from web based software GOEAST [47]. In addition, the embeded tool of Multi-GOEAST was used to compare the difference of the GO terms that were enriched in these two sets of genes.

### MiRNA target prediction

Pre-compiled predicted miRNA targets were retrieved from previously constructed databases including TargetScan (http://www.targetscan.org/, release 5.1: April 2009), PicTar (from UCSC table browser, http://genome.ucsc.edu/) and miRanda (http://www.microrna.org/, August 2010). These algorithms are considered as having high accuracy for miRNA target prediction [48,49]. The intersection dataset generated by both TargetScan and PicTar were retrieved from miRGen database. We also included another dataset generated from PITA software (from the Weizman Institute website, http://genie.weizmann.ac.il/pubs/mir07/mir07_data.html, no flank, TOP catalog), which makes predictions based only on sequence features and target site accessibility. Experimentally validated miRNA targets were integrated from miRTarBase http://mirtarbase.mbc.nctu.edu.tw/, miRrecords http://mirecords.umn.edu/miRecords, miRWalk http://mirwalk.uni-hd.de/ and miR2Disease http://www.miR2Disease.org.

### Computational framework

Three different methods were used to analyze the influence of miRNAs on gene expression fluctuation. Firstly, we calculated the proportion of predicted miRNA targets among SE genes and FL genes at different level of significance. As a control, the same numbers of genes were randomly selected from the gene list and the proportion of miRNA targets among these genes was calculated. Secondly, to avoid potential sampling bias, we divided the total genes into two distinct groups. The first group contained the union of the predicted miRNA targets (predicted to be a target by at least one method), whereas the second group contained all of the non-miRNA targets, i.e., the genes that were not predicted to be a target by any of these prediction tools. The average FL score from different groups was calculated to compare the differences. Lastly, we used a sliding window method to calculate the correlation between the average FL score and the proportion of miRNA targets. Specifically, genes were rank ordered according to their FL scores, the average FL score and the miRNA target proportion was calculated for the top 2,000 genes ( = window size) in the first group, then the window was shifted by 50 genes ( = step size) to perform the same calculation on the next group until the end. Pearson's correlation coefficient was calculated between the average FL score and the miRNA target proportion from different groups.

## Competing interests

The authors declare that they have no competing interests.

## Authors' contributions

ZY carried out the data analysis and drafted the manuscript. DD participated in the design of the study and in drafting the manuscript. MJCC participated in the data analysis and revised the manuscript. LW participated in the data analysis. ZZ and YZ conceived the study and helped to revise the manuscript. All authors read and approved the final manuscript.

## Supplementary Material

Additional file 1 **Figure S1: GO term distribution of SE genes and FL genes (molecular function)**. The enriched GO terms were colored red for SE genes and green FL genes. A distinct GO term distribution of molecular function for the two sets of genes was observed. SE genes were mainly enriched in RNA binding, protein binding, NADH dehydrogenase activity and constituent of ribosome etc, whereas FL genes were mainly enriched in the receptor binding, cytokine activity, growth factor receptor binding, peptide hormone binding and dopamine binding etc. Click here for file

Additional file 2 **Figure S2: GO term distribution of SE genes and FL genes (biological process)**. The enriched GO terms were colored red for SE genes and green FL genes. A distinct GO term distribution of biological process for the two sets of genes was observed. SE genes were mainly enriched in translation, gene expression, macromolecule metabolic, biosynthetic etc, whereas FL genes were mainly enriched in signaling pathways, defense response, regulation of immune system process and mediated by a chemical signal etc. Click here for file

Additional file 3 **Figure S3: miRNA targets are not enriched in control group**. This figure shows the number of miRNA targets and non-miRNA targets among control group predicted (A) by PicTar, (B) by TargetScan, (C) by both PicTar and TargetScan (intersections), (D) by PITA, (E) by miRanda and (F) by experimentally validated miRNA targets when 5% of the genes were randomly designated as SE genes and FL genes respectively. Click here for file

Additional file 4 **Figure S4: miRNA targets are enriched in SE genes (top 10%)**. This figure shows the number of miRNA targets and non-miRNA targets among SE genes and FL genes predicted (A) by PicTar, (B) by TargetScan, (C) by both PicTar and TargetScan (intersections) and (D) by PITA, (E) by miRanda and (F) by experimentally validated miRNA targets when top and bottom 10% of the gene designated as SE genes and FL genes respectively. Click here for file

Additional file 5 **Figure S5: miRNA targets are enriched in SE genes derived only from normal tissues**. This figure shows the number of miRNA targets and non-miRNA targets among SE genes and FL genes predicted (A) by PicTar, (B) by TargetScan, (C) by both PicTar and TargetScan (intersections) and (D) by PITA, (E) by miRanda and (F) by experimentally validated miRNA targets when top and bottom 5% of the genes derived only from normal tissues designated as SE genes and FL genes respectively. Click here for file

Additional file 6 **Figure S6: correlation between gene expression fluctuation and number of regulatory miRNAs**. No obvious correlation between expression fluctuation and number of regulatory miRNAs was observed. (A) average FL score and number of regulatory miRNAs from PicTar results, Pearson correlation coefficient, r = 0.16, p value: 0. 24. (B) average FL score and number of regulatory miRNAs from TargetScan results, Pearson correlation coefficient, r = 0.10, p value: 0.49. (C) average FL score and number of regulatory miRNAs from PITA results, Pearson correlation coefficient, r = 0.124, p value: 0.59. Click here for file

Additional file 7 **Table S1: retrieved SE-miRNAs and FL-miRNAs**. This table lists the miRNA ID and number of targets in both SE genes and FL genes predicted by PicTar, TargetScan and PITA. The p value were inferred from Fisher exact test. Click here for file

Additional file 8 **Table S2: microarray data sets used for this analysis**. This table lists the GEO ID, brief description, number of samples and sample type of 149 microarray data sets used for this analysis, which includes 69 data sets from normal tissue, 59 data sets from cancer tissue or cell line and 21 data sets from other disease. Click here for file

Additional file 9 **Table S2: retrieved SE genes and FL genes and their FL Scores**. This table lists the SE genes and FL genes obtained from 149 microarray data sets and from 69 microarray data sets based on normal tissues respectively. Click here for file
