# Supplementary figures and images for: Preferential regulation of stably expressed genes in the human genome suggests a widespread expression buffering role of microRNAs
Source: BMC Genomics. 2012 Dec 7;13(Suppl 7):S14. doi: 10.1186/1471-2164-13-S7-S14 (PMC3521228; doi:10.1186/1471-2164-13-S7-S14)

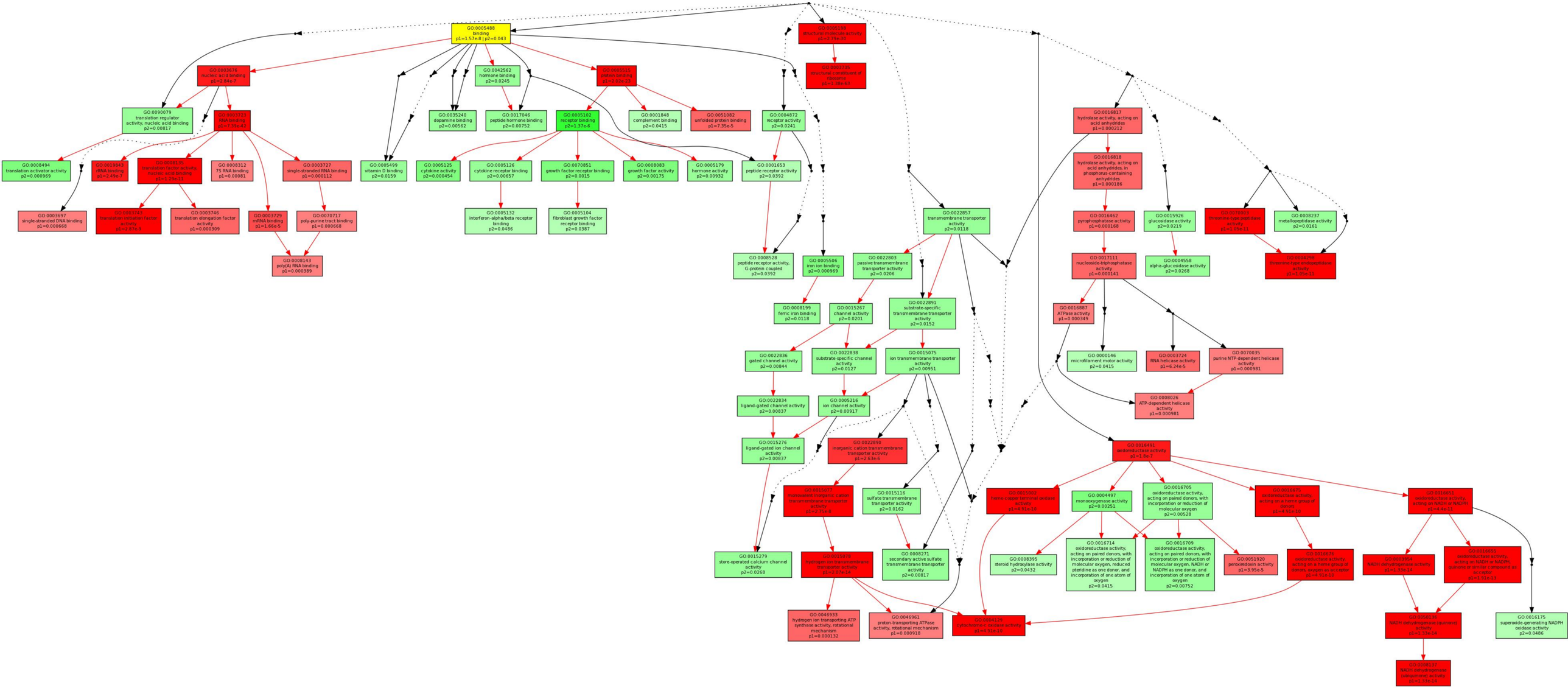

Supplement: Additional file 1 — Figure S1: GO term distribution of SE genes and FL genes (molecular function). The enriched GO terms were colored red for SE genes and green FL genes. A distinct GO term distribution of molecular function for the two sets of genes was observed. SE genes were mainly enriched in RNA binding, protein binding, NADH dehydrogenase activity and constituent of ribosome etc, whereas FL genes were mainly enriched in the receptor binding, cytokine activity, growth factor receptor binding, peptide hormone binding and dopamine binding etc. [file 1471-2164-13-S7-S14-S1.pdf]

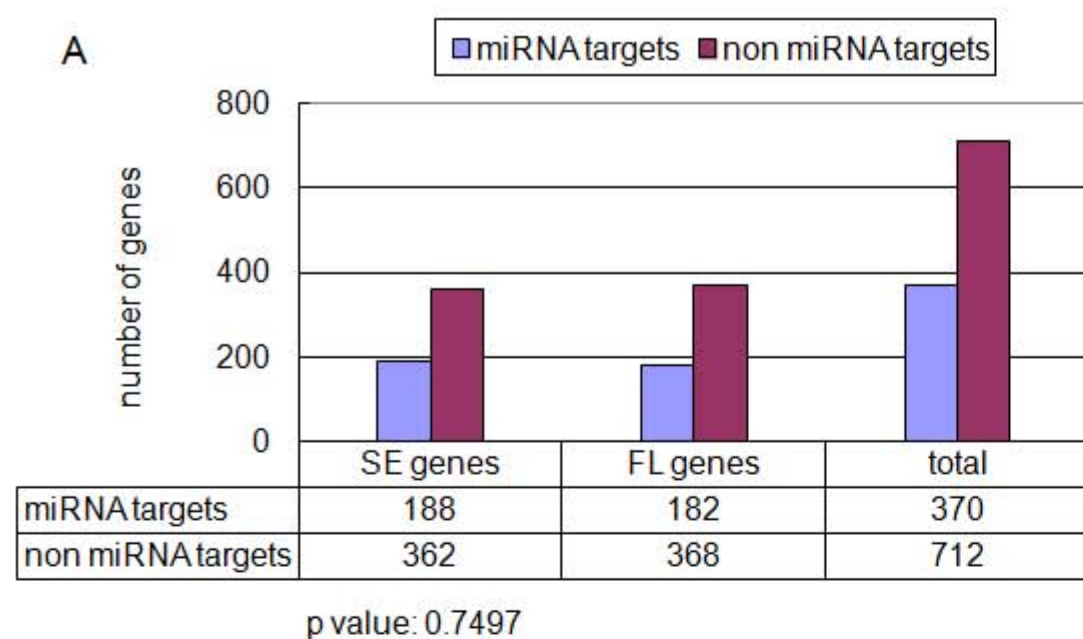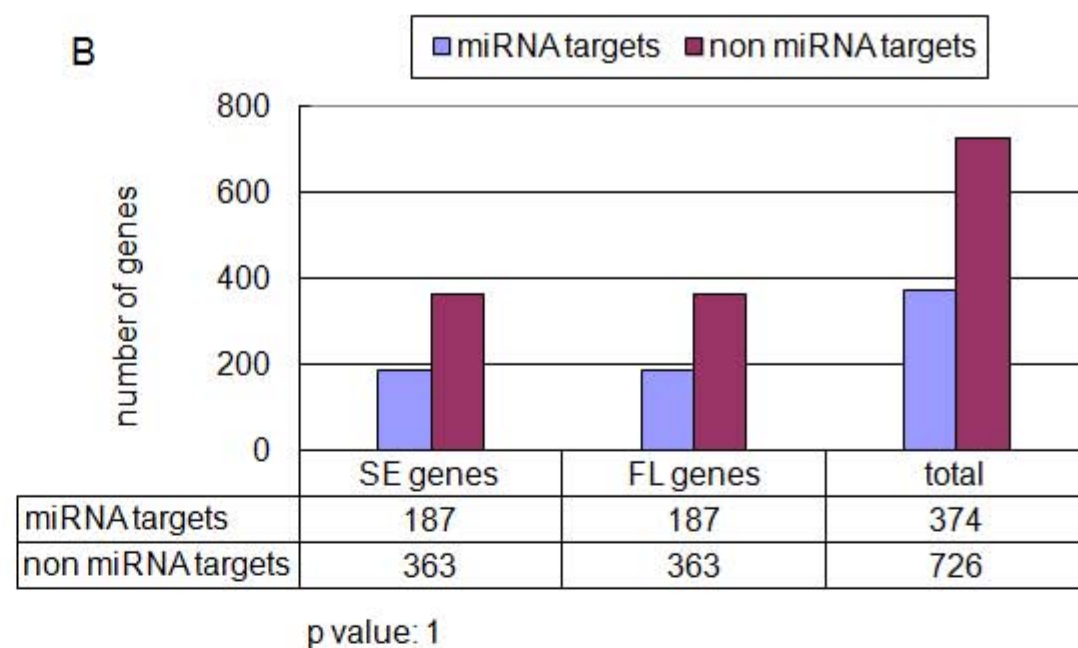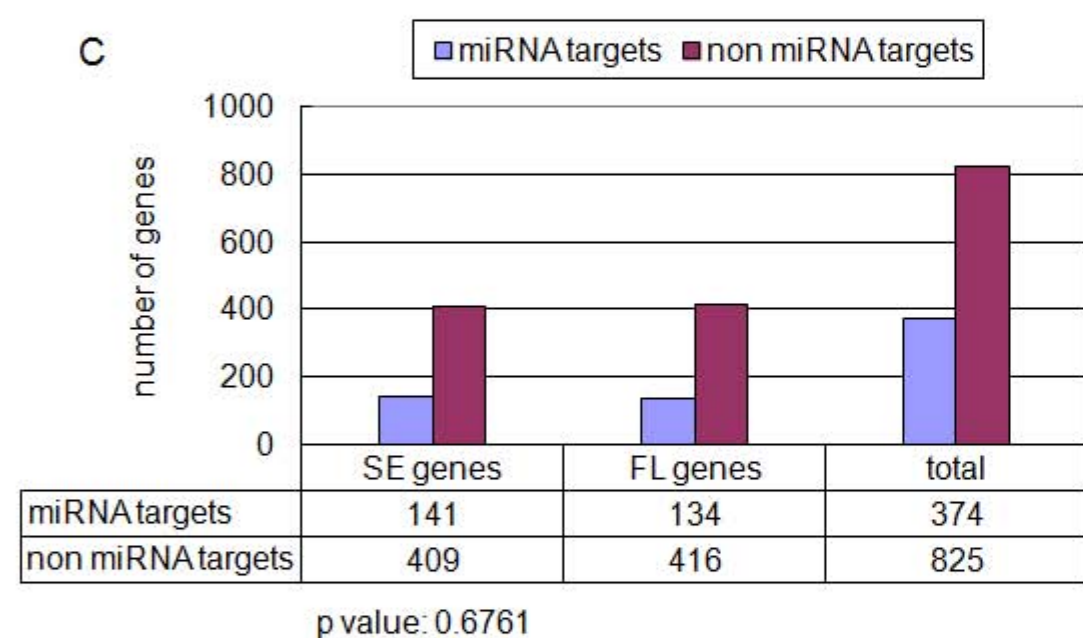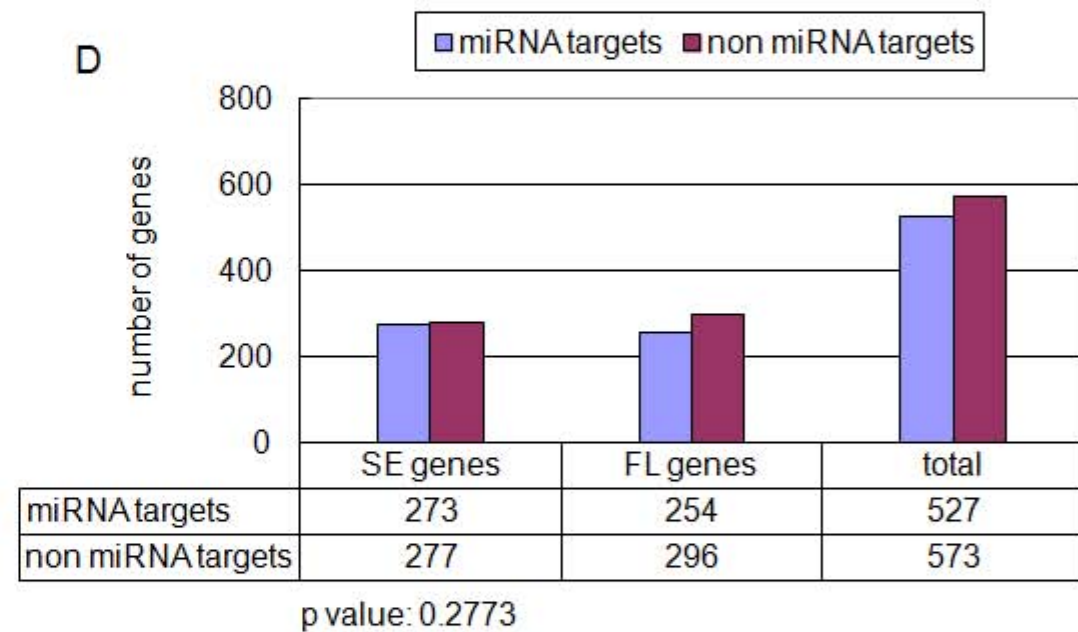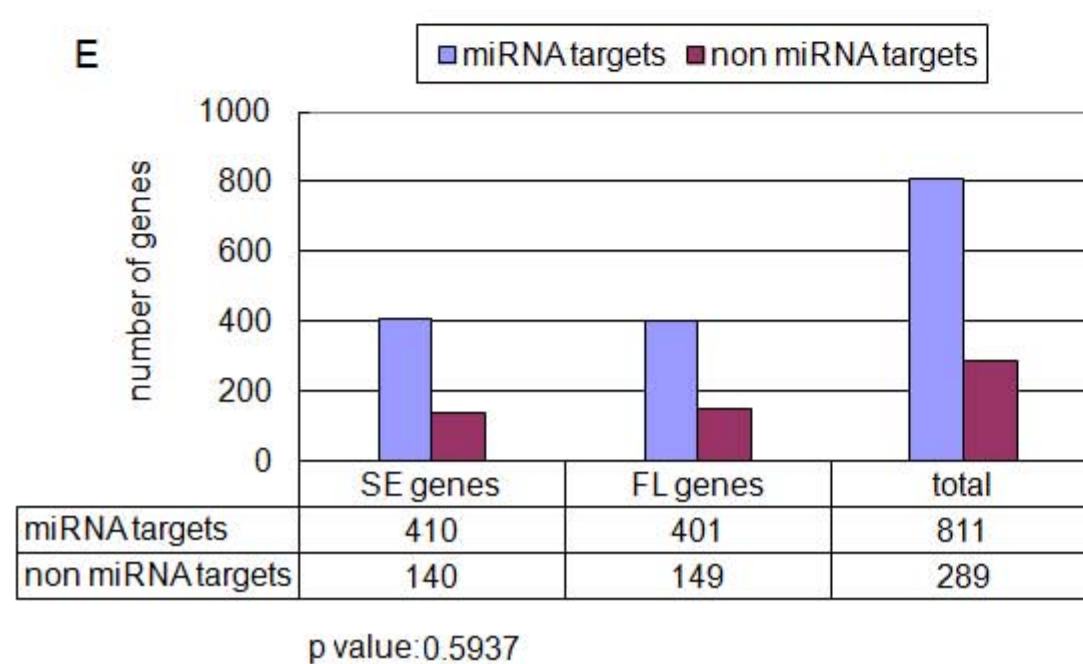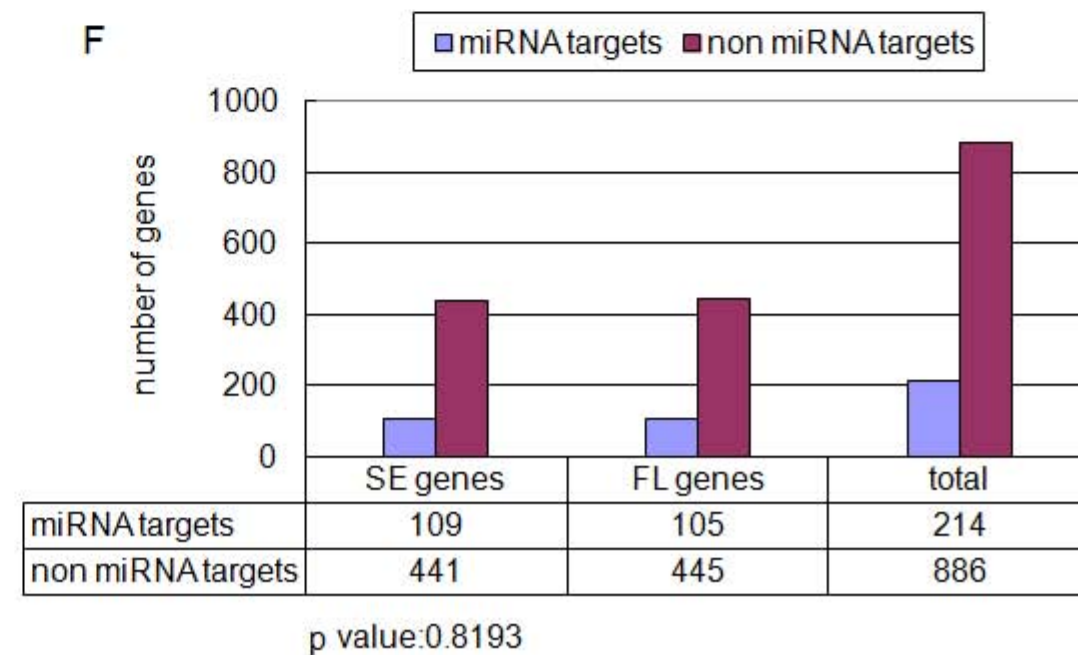

Supplement: Additional file 3 — Figure S3: miRNA targets are not enriched in control group. This figure shows the number of miRNA targets and non-miRNA targets among control group predicted (A) by PicTar, (B) by TargetScan, (C) by both PicTar and TargetScan (intersections), (D) by PITA, (E) by miRanda and (F) by experimentally validated miRNA targets when 5% of the genes were randomly designated as SE genes and FL genes respectively. [file 1471-2164-13-S7-S14-S3.pdf]

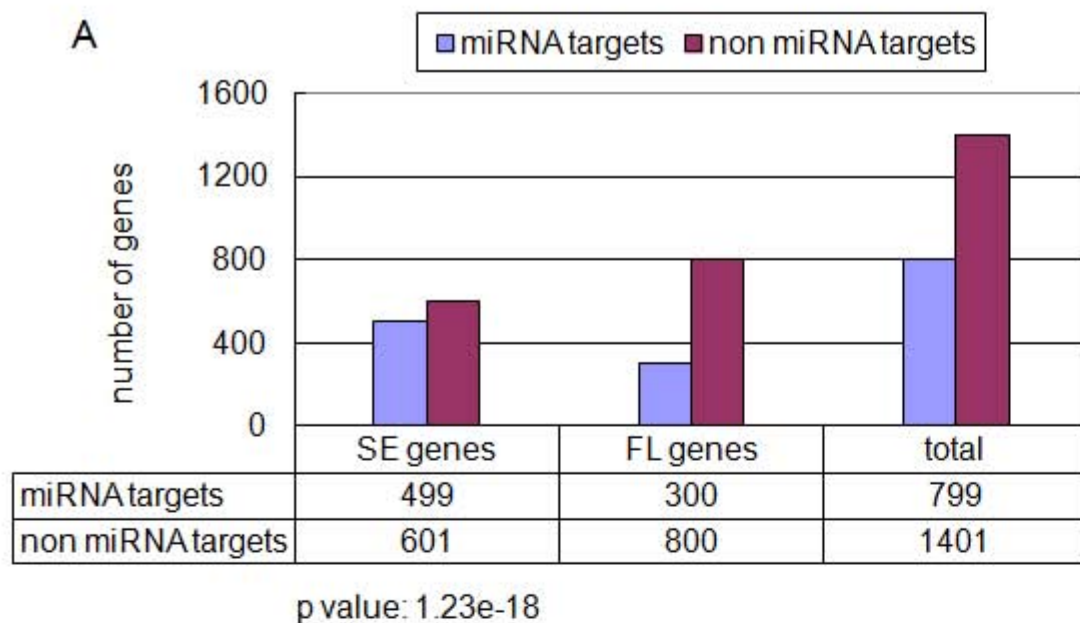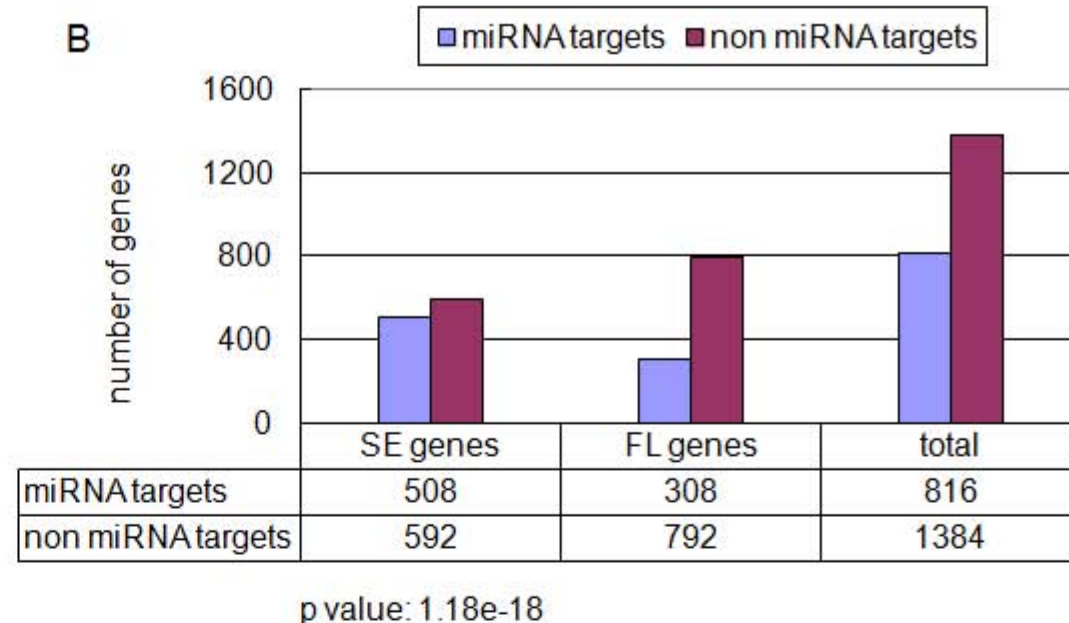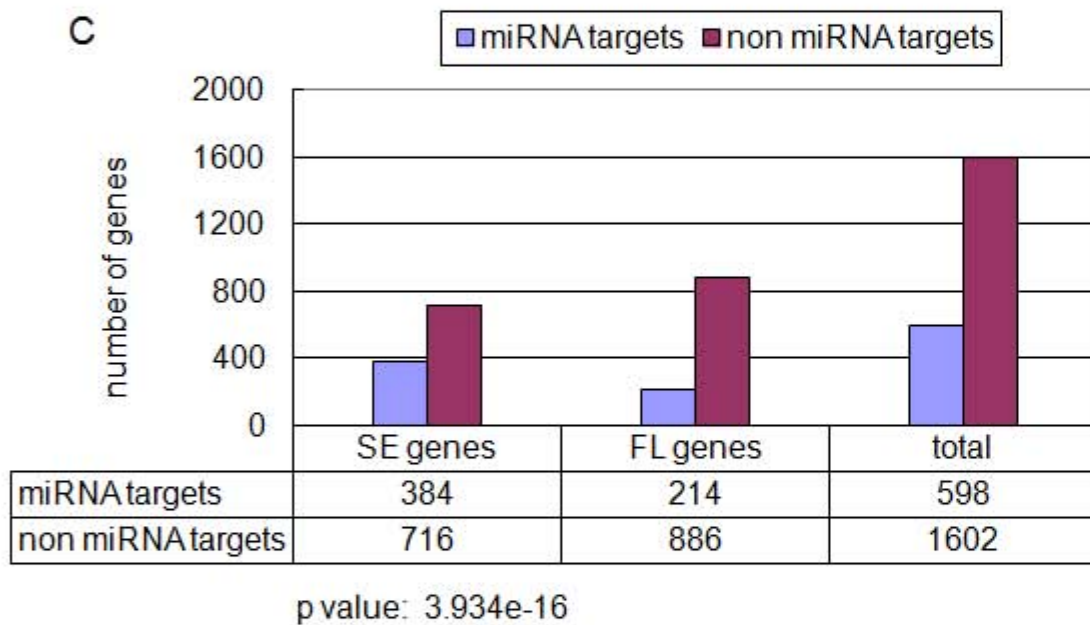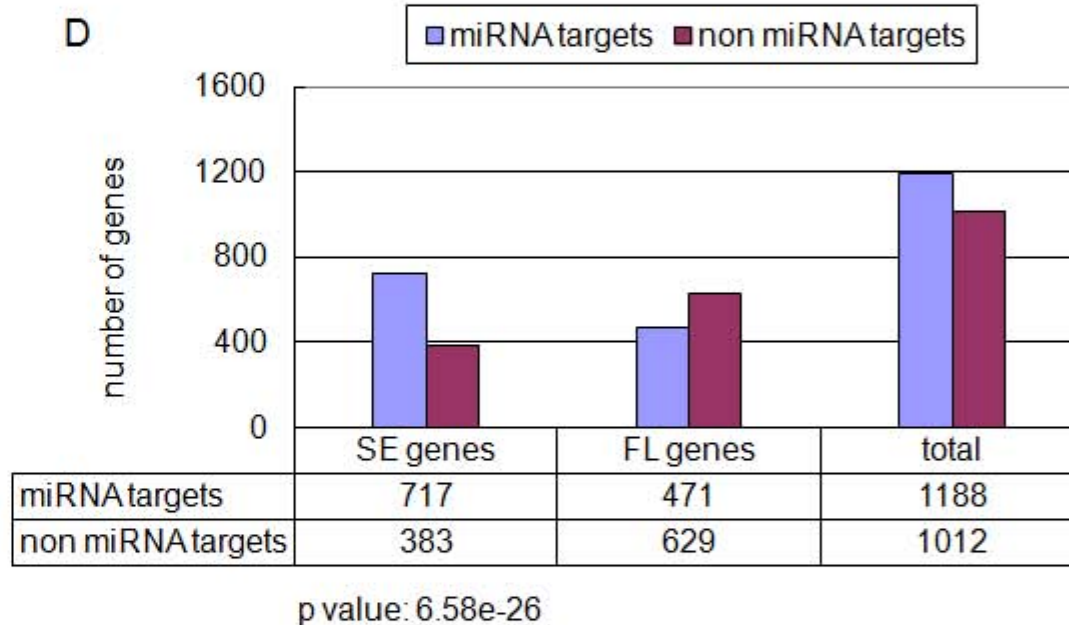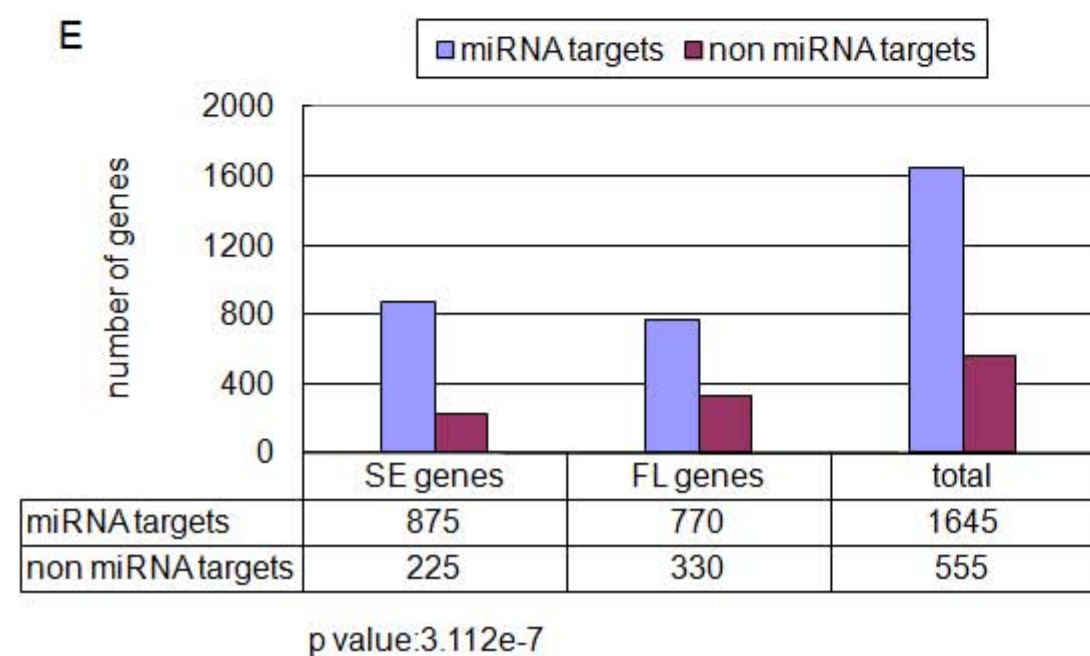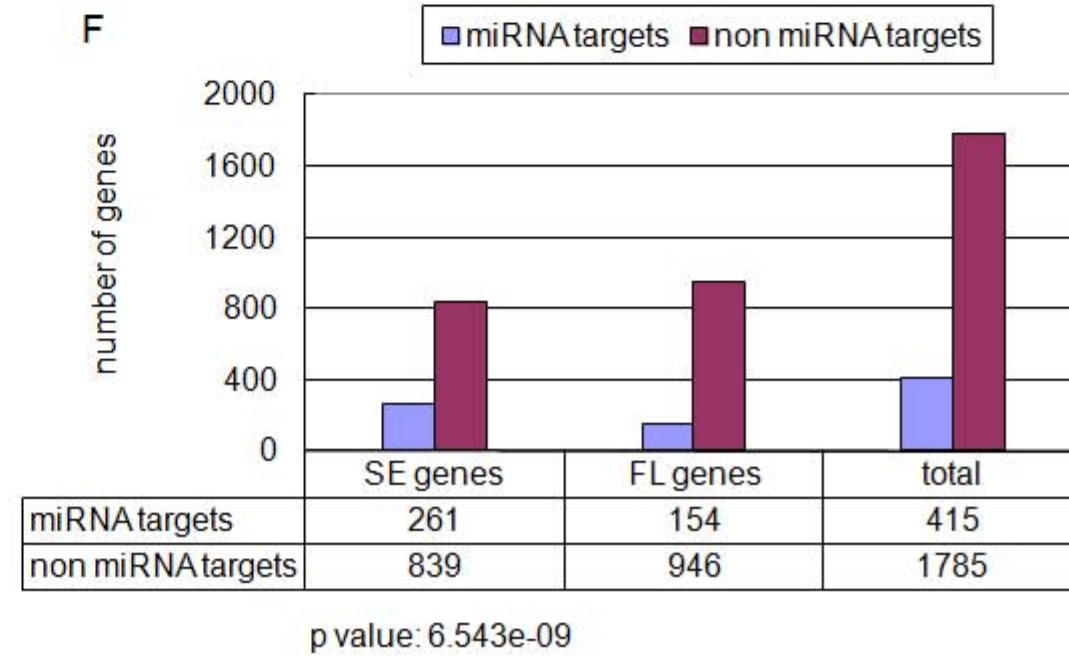

Supplement: Additional file 4 — Figure S4: miRNA targets are enriched in SE genes (top 10%). This figure shows the number of miRNA targets and non-miRNA targets among SE genes and FL genes predicted (A) by PicTar, (B) by TargetScan, (C) by both PicTar and TargetScan (intersections) and (D) by PITA, (E) by miRanda and (F) by experimentally validated miRNA targets when top and bottom 10% of the gene designated as SE genes and FL genes respectively. [file 1471-2164-13-S7-S14-S4.pdf]

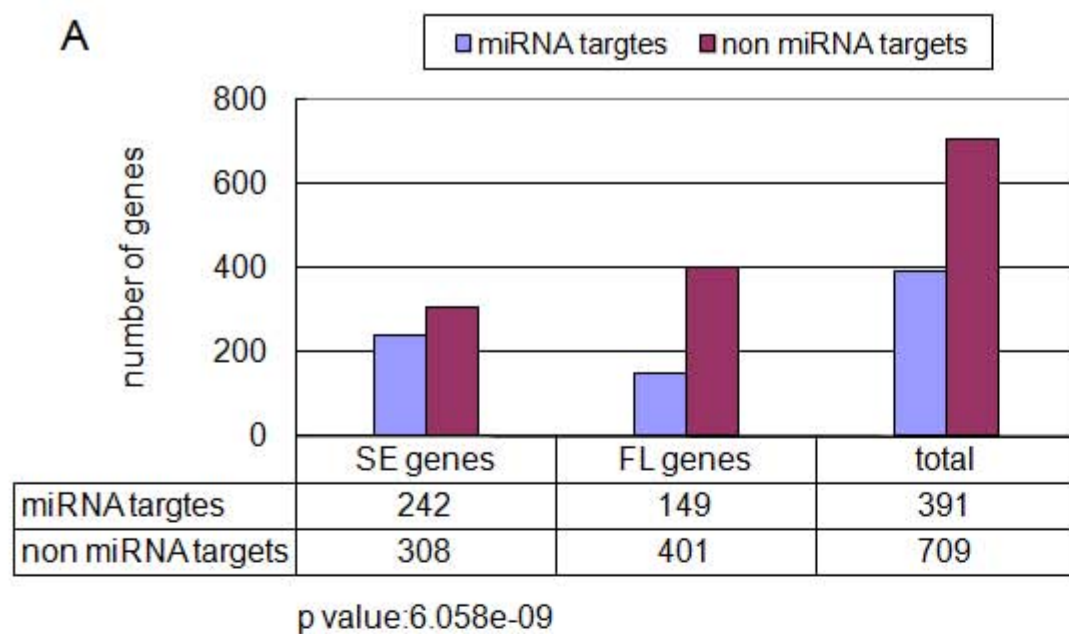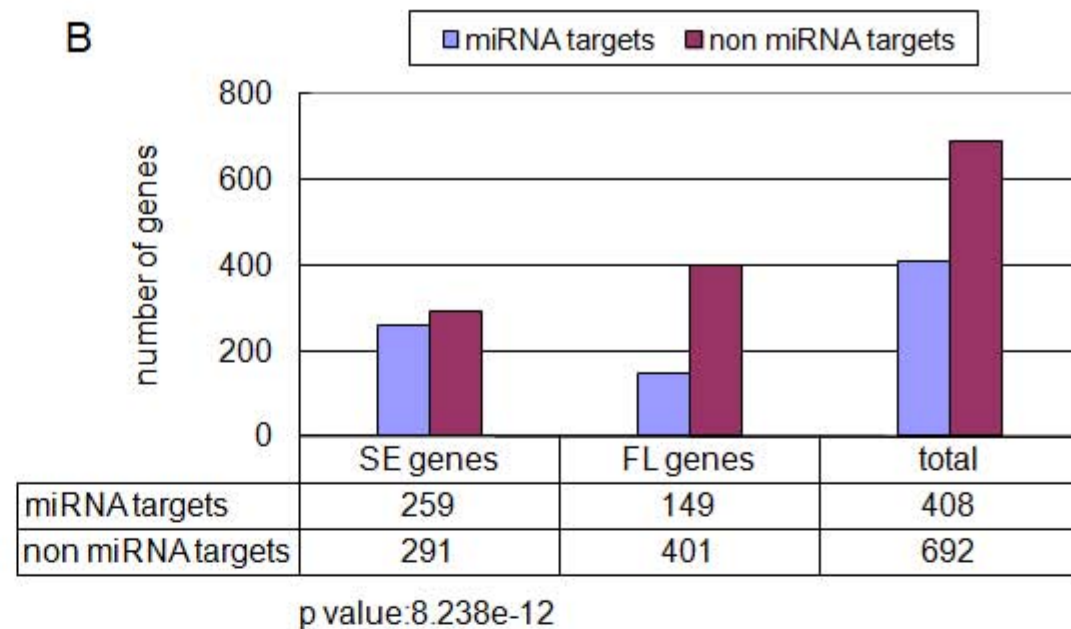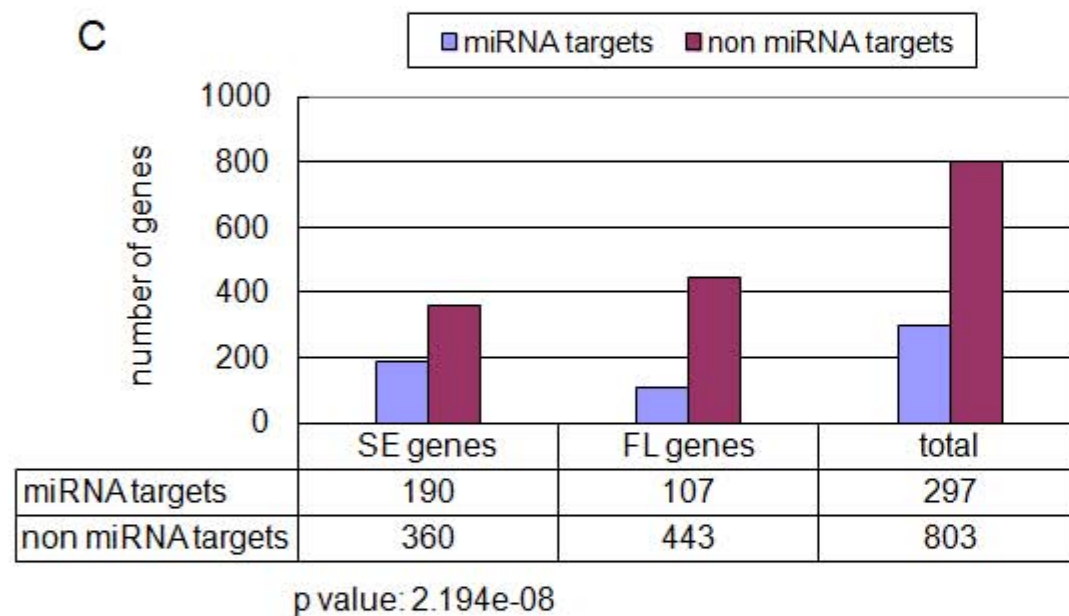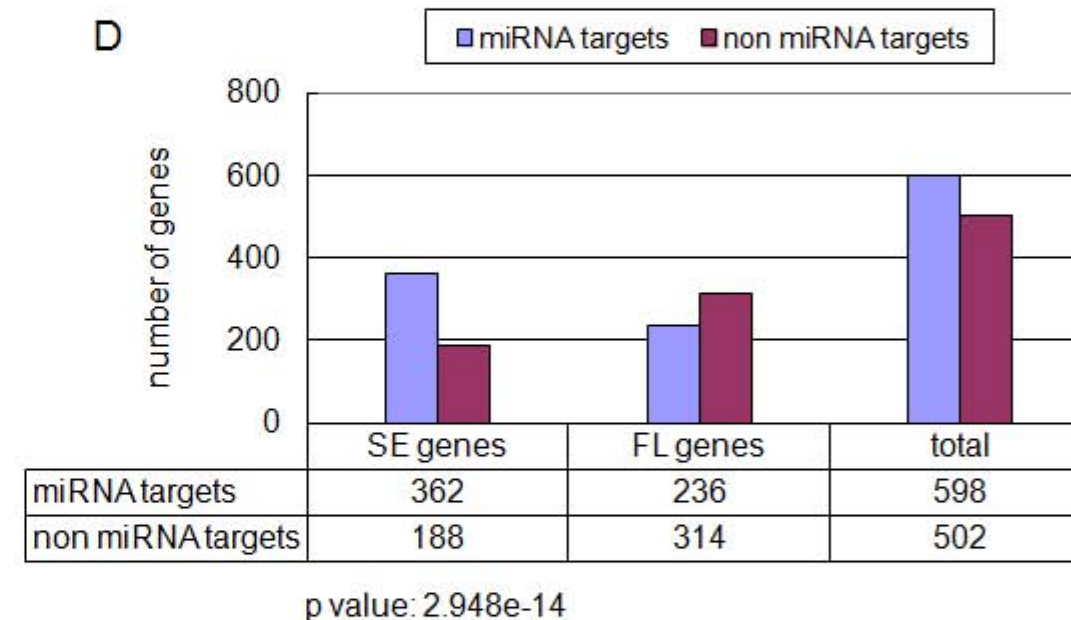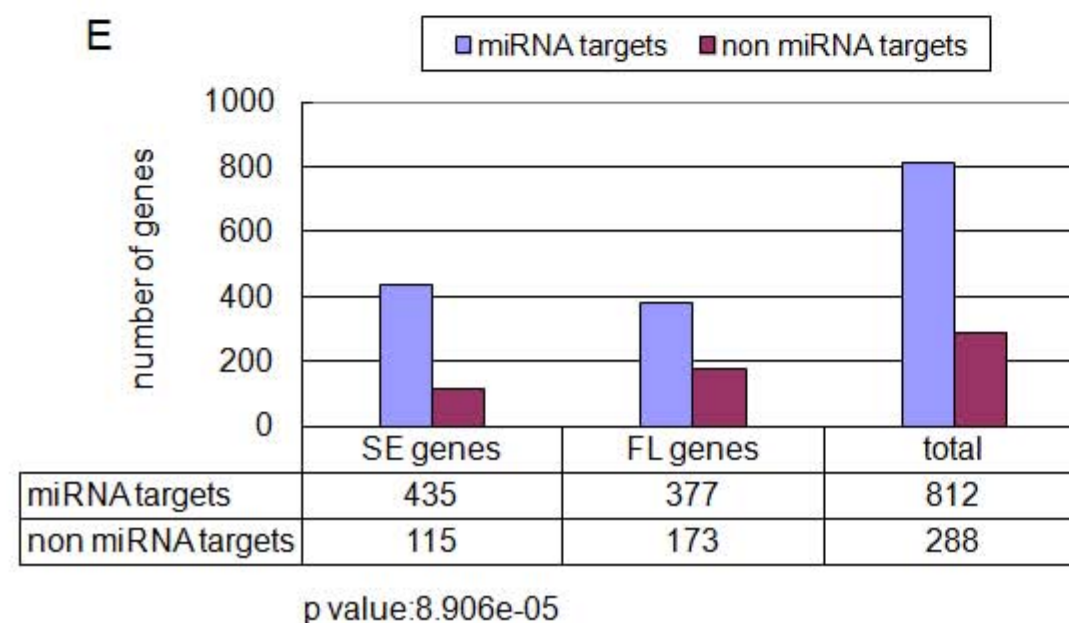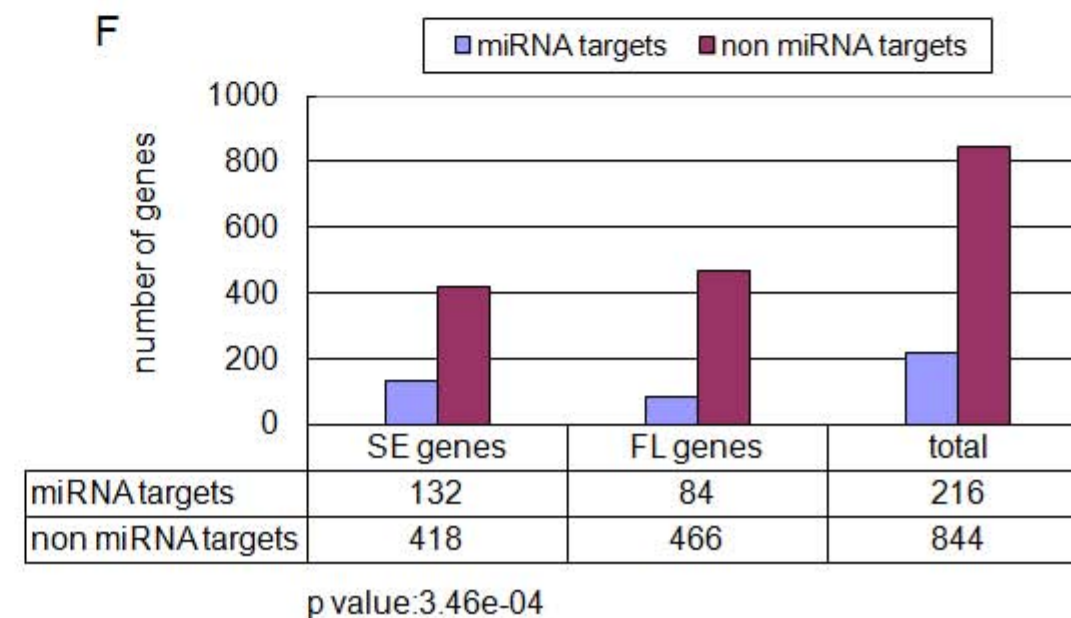

Supplement: Additional file 5 — Figure S5: miRNA targets are enriched in SE genes derived only from normal tissues. This figure shows the number of miRNA targets and non-miRNA targets among SE genes and FL genes predicted (A) by PicTar, (B) by TargetScan, (C) by both PicTar and TargetScan (intersections) and (D) by PITA, (E) by miRanda and (F) by experimentally validated miRNA targets when top and bottom 5% of the genes derived only from normal tissues designated as SE genes and FL genes respectively. [file 1471-2164-13-S7-S14-S5.pdf]

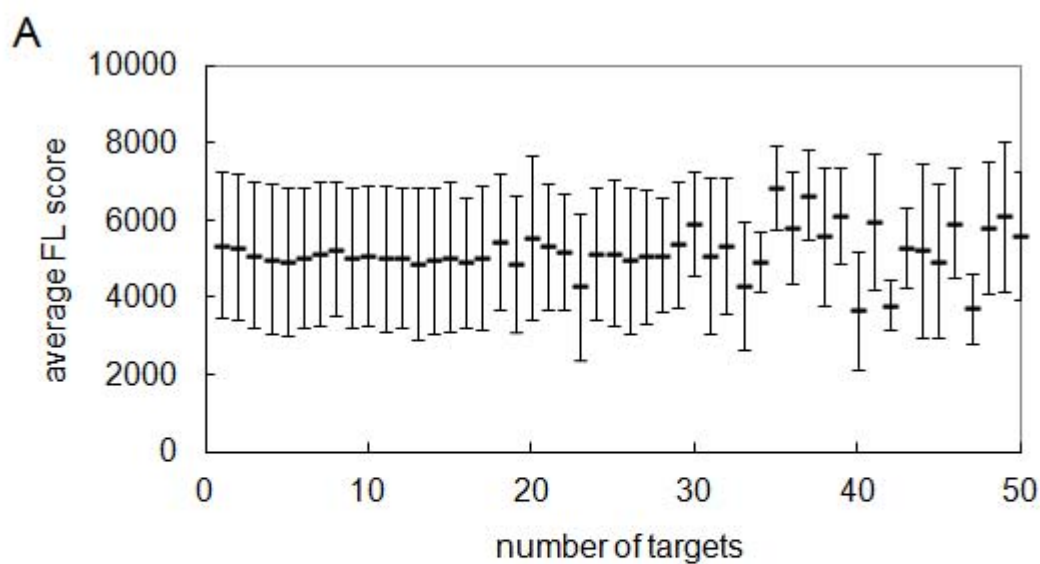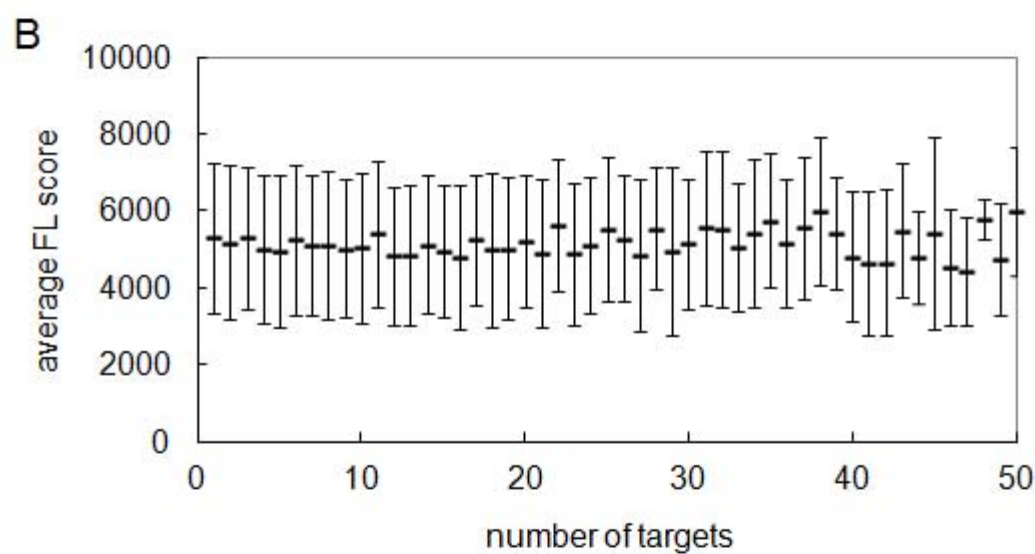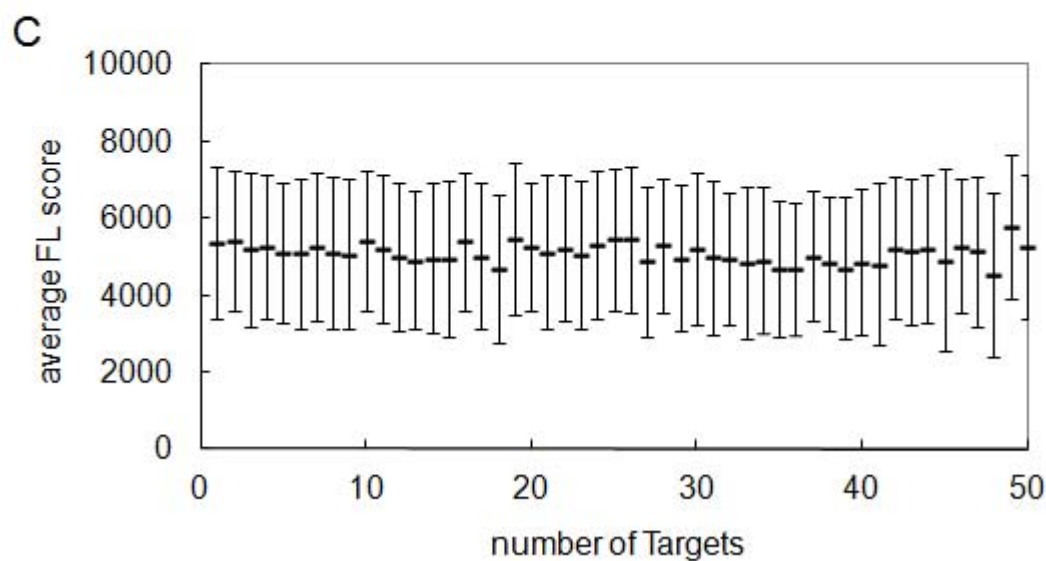

Supplement: Additional file 6 — Figure S6: correlation between gene expression fluctuation and number of regulatory miRNAs. No obvious correlation between expression fluctuation and number of regulatory miRNAs was observed. (A) average FL score and number of regulatory miRNAs from PicTar results, Pearson correlation coefficient, r = 0.16, p value: 0. 24. (B) average FL score and number of regulatory miRNAs from TargetScan results, Pearson correlation coefficient, r = 0.10, p value: 0.49. (C) average FL score and number of regulatory miRNAs from PITA results, Pearson correlation coefficient, r = 0.124, p value: 0.59. [file 1471-2164-13-S7-S14-S6.pdf]
