# Supplementary material for: Preferential regulation of stably expressed genes in the human genome suggests a widespread expression buffering role of microRNAs
Source: BMC Genomics. 2012 Dec 7;13(Suppl 7):S14. doi: 10.1186/1471-2164-13-S7-S14 (PMC3521228; doi:10.1186/1471-2164-13-S7-S14)

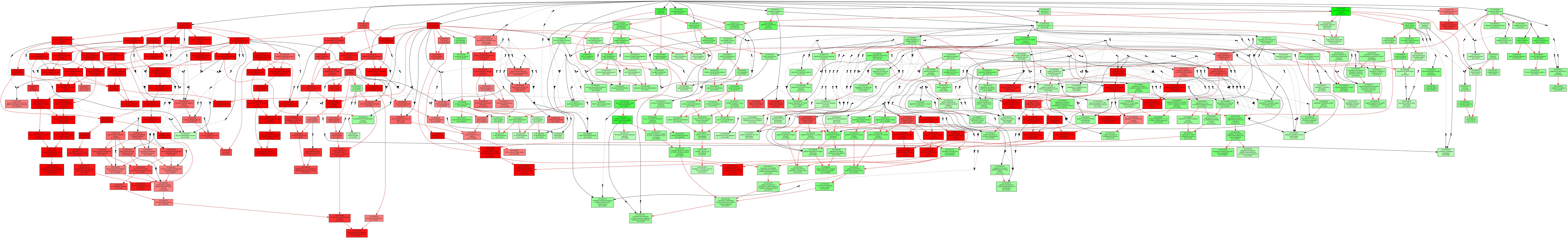

Supplement: Additional file 2 — Figure S2: GO term distribution of SE genes and FL genes (biological process). The enriched GO terms were colored red for SE genes and green FL genes. A distinct GO term distribution of biological process for the two sets of genes was observed. SE genes were mainly enriched in translation, gene expression, macromolecule metabolic, biosynthetic etc, whereas FL genes were mainly enriched in signaling pathways, defense response, regulation of immune system process and mediated by a chemical signal etc. [file 1471-2164-13-S7-S14-S2.pdf]
